# Working Women in Large Towns

**Published:** 1890-10-18

**Authors:** 


### Working Women in Large Towns.

### XIII.—SOME SOURCES OF DISTRESS.

*Working for Pocket Money.*—It is well known that many women and girls work, not for their living, but for money to be spent on dress and little extras, their parents, or it may be their husbands, supplying them with board and lodging. They are able and willing, therefore, to take work at a price which could not possibly give them a fair day's wage—that is, a wage which they could live upon; and thus they bring untold distress upon such of their sisters as have no other means of subsistence—for why should the employer pay more to a poor friendless girl than the daughter of a small tradesman will take for exactly the same work? They themselves may be unconscious of the harm they are doing; but the poor workers whom they undersell are quite aware of it, and feel it bitterly. "Of course," says a poor mantle-liner, after giving particulars of the wretched price paid for her work, "of course it's too little—starvation wages; but I suppose the 'boss' can't help it. There are a lot of young women with clerks for husbands, he tells me, that go to him and beg for work at any price, just to get pocket-money for themselves. He says it is a favour to me to let me have any, although I have been working for him for years." What is wanted here is that someone should put the facts of the case sensibly and strongly before these "pocket-money workers," showing them the harm which they unwittingly bring upon their poorer sisters; asking them why their fathers, just because they happen to earn a good wage, should make a present of part of it to their daughters' employers; pointing out to them that if sudden reverses forced them to depend on themselves, they would bitterly regret the loss of the money which should in fact have been theirs; pressing upon them the desirability, both for themselves and for others, of combining together, so as to force wages up to their proper level. Among girls of the poorer middle classes this is specially important; for there is in these classes a large preponderance of unmarried women over unmarried men; and the permanent depression of women's wages must in the end mean to many women but a choice between distressing poverty and a life of shame.

*Bad Work.*—We decided at first upon the heading Want of Skill, but as the alternative title is the more comprehensive of the two, we have adopted it. Our pity for the poor underpaid workers in shirtmaking and other home industries must not lead us to forget that the sins and frailties of human nature are to be found in their class as well as in that of the employers. They are often careless, unbusiness-like, and undependable, will scamp work where they can, and try to "best" their employers, whether by fair means or foul. That they have more excuse than many of us who do the like does not alter the fact that they themselves are in part responsible for their miseries. True, all low-class work, especially low-class work done at home, would seem to necessitate the presence of the sweater, for someone there must be who will undertake the little individual transactions which a larger man cannot give time to, and be responsible not only for the work done, but for the safety of the materials given out. But, as his profits must come out of their wages, it is obviously to their advantage to diminish, by means of good and honest work, his risks and his labour. Moreover, experience teaches that dependable workers may count on being recognised by employers, at any rate in the long run, and so, in various ways, reap the benefit of their carefulness and honesty.

With regard to want of skill, this is too often the misfortune, rather than the fault, of the worker. But we have such overwhelming evidence to prove that unskilled labour and miserable wages always go hand in hand—at least, in this highly-civilised country of ours, that every effort should be made to impress this fact upon the poor, and upon those who are interested in them. It is a great question whether, as things are, very poor work can possibly be paid for at a rate which will keep the worker in even the barest necessaries of life. Such work is, in fact, a mistake altogether. "While the worker is starved, the consumer is defrauded," says Miss Potter, of the lowest class of tailoring; and if no one profits by it, clearly the less we have of such work the better. It is impossible, alas! to do much for those poor women who have never been taught a trade, who toil on day after day at "slop-work," and whose life, as Miss Collet but too truly puts it, is but procrastination of death. But all our energies should be brought to bear upon the question of improved technical training for girls. To teach them not only trades, but the rudiments of domestic work, would give them an enormous lift in the struggle of life; work would be better done, wages would be raised, English workers would hold their own against foreign competition, and many a problem, hopeless at present, might become capable of solution. Many technical schools for women exist in France; why should they not be started in England? We by no means intend to disparage the efforts which have already been made in this direction; but we do say that they are a mere nothing as compared with the need.

One is sometimes tempted to wonder what would be the permanent effect upon wages of a great increase in the number of women—or men—able to undertake skilled labour; whether the competition between them would not in time become almost as keen as that which exists now among unskilled workers, until wages were again reduced to their former low level. But this is another of those questions which are theoretically, not practically, important. We will only express our belief that, for reasons which cannot be given here, the answer would be a hopeful one; and remark that, as far as regards our own country and its relations with others, an increase of skill can be productive of nothing but good, by enabling us to compete more successfully with foreign workers. As for the social and moral advantages of increased skill among our workers, we will speak of them later on.

It is only right to mention, as being somewhat against our view of the question, that in certain trades—mantle-lining, for example—skilled workers are sometimes paid at a very low rate, and that they might actually earn more than they do if they were willing to take up a lower class of work, either in their own trade or in another. But we have good authority for believing that this curious state of things is not only exceptional, and owing to accidental circumstances, but that it might be remedied by proper and united action on the part of the workers; and we therefore feel justified in paying but little attention to a fact which at first sight seemed to invalidate our conclusion.

